# Amyloidosis with Cardiac Involvement: Identification, Characterization, and Management

**DOI:** 10.1007/s11899-021-00626-4

**Published:** 2021-06-09

**Authors:** Faizi Jamal, Michael Rosenzweig

**Affiliations:** 1 Department of Medicine, Division of Cardiology, City of Hope, Duarte, CA USA; 2 Department of Hematology, City of Hope, 1500 E Duarte Rd. Duarte, CA, Duarte, CA 91010 USA

**Keywords:** Amyloid, Transthyretin, Cardiomyopathy, Light chain, Pyrophosphate, Congo red

## Abstract

**Purpose of Review:**

Amyloidosis is a protein deposition disease whereby a variety of precursor proteins form insoluble fibrils that deposit in tissues, causing organ dysfunction and, many times, death. Accurate characterization of the disease based on the nature of the precursor protein, organ involvement, and extent of disease is paramount to guide management. Cardiac amyloidosis is critical to understand because of its impact on prognosis and new treatment options available.

**Recent Findings:**

New imaging methods have proven to be considerably valuable in the identification of cardiac amyloid infiltration. For treating clinicians, a diagnostic algorithm for patients with suspected amyloidosis with or without cardiomyopathy is shown to help classify disease and to direct appropriate genetic testing and management. For patients with light chain disease, recently introduced treatments adopted from multiple myeloma therapies have significantly extended progression-free and overall survival as well as organ response. In addition, new medical interventions are now available for those with transthyretin amyloidosis.

**Summary:**

Although cardiac amyloidosis contributes significantly to the morbidity and mortality associated with systemic disease, new tools are available to assist with diagnosis, prognosis, and management.

## Introduction

Amyloidosis is a collection of diseases characterized by the progressive extracellular deposition of insoluble fibrillar proteins in tissues, resulting in organ dysfunction, and, many times, death [1]. All amyloid deposits have a similar structure consisting of anti-parallel β-strands, measuring 7–13 nm in diameter and forming the characteristic beta pleated sheets [2, 3]. In addition to fibrils, amyloid deposits contain non-fibrillary components including glycosaminoglycans (GAGs) and serum amyloid P component (SAP) [4, 5]. The specific ultrastructure of amyloid fibrils accounts for their pathognomonic property of binding Congo red dye in a spatial manner that produces green birefringence when viewed under polarized light [6].

The diseases are highly varied; therefore, accurate description and characterization are critical. Cardiac involvement from amyloid deposition is relatively common and a major cause of morbidity and mortality. In this review, the major subtypes of amyloid will be examined, as well as a discussion on amyloid-related heart disease with updates on diagnostic tools and management options.

## Classification

Amyloidosis is first categorized as either systemic or localized. In localized disease, amyloid fibrils composed of light chains deposit in just one organ because of in situ production of amyloidgenic protein by a population of clonal B cells in the affected tissues. Common sites of localized disease include the bladder, respiratory tract, and skin. Localized amyloidosis can also involve the gastrointestinal tract and needs to be carefully discerned from systemic disease [7]. Localized amyloidosis is frequently indolent and managed with local surgical measures as well as radiation in rare, select cases [8]. Although localized disease almost never evolves into systemic disease, patients should be monitored after local management for progressive disease or recurrence [9]. Conversely, systemic disease is due to systemic production of amyloidgenic proteins. Multiple organs are either involved at the time of diagnosis or at risk for progressive involvement over time.

Amyloidosis is further classified by the nature of the innate precursor protein that misfolds to form the fibrillar deposits. The major types of amyloidosis are light chain (AL), transthyretin-derived (ATTR) amyloidosis, and secondary amyloidosis (AA). There are now more than 30 proteins known to form amyloid in humans [4]. Systemic amyloidosis can either be acquired or inherited. Acquired disease can develop with the onset of underlying plasma cell dyscrasia, uncontrolled inflammation, or advanced age, as seen in AL, AA, or wild-type transthyretin disease (wtATTR), respectively. Alternatively, systemic amyloidosis may develop because of an inherited mutation of the TTR gene (hATTR) or another less common genetic mutation [10].

Finally, the nature and degree of organ involvement further define a patient’s illness, with impact on initial presentation, management, and prognosis. Amyloid fibrils from a systemic process can deposit anywhere in the body except the central nervous system. Clinical features are non-specific, mimicking more common disease presentations, often leading to a delay in diagnosis. The kidneys are commonly involved in several forms of systemic disease. Albuminuria and nephrotic syndrome are the classic presentations, but renal dysfunction may also be present. Cardiac involvement occurs in about 50% of patients and is the leading cause of morbidity and mortality [11]. Amyloid infiltration of the heart typically presents as a restrictive cardiomyopathy. In addition to infiltrative heart disease, the circulating precursor light chain in AL amyloidosis has direct cardiac toxicity. Improvement in cardiac function in preclinical models and clinical experience are noted with a drop in light chain concentration [12, 13]. Peripheral motor sensory as well as autonomic neuropathy is a common feature of AL amyloidosis as well as hereditary subtypes. Small fiber-mediated sensations of heat or cold are often the initial manifestation and easily misattributed as more common etiologies. Autonomic neuropathy can be particularly debilitating, resulting in erectile dysfunction as well as postural hypotension, early satiety, and diarrhea and/or constipation. Liver involvement often presents as hepatomegaly with an elevated alkaline phosphatase. Soft tissue deposition including macroglossia and periorbital ecchymosis as well as salivary and sub-mandibular lymph node infiltration are almost unique to AL amyloidosis [6]. Carpal tunnel syndrome, also due to soft tissue involvement, is an early often unrecognized symptom in AL as well as TTR amyloidosis. A 10.2% rate of amyloidosis was described in a prospective cohort of men over 50 and women over 60 undergoing surgery for idiopathic, bilateral carpal tunnel syndrome [14]. Common subtypes of systemic amyloidosis are shown [6] (Table 1).

**Table 1:** Systemic amyloidosis subtypes

Amyloid type	Acquired or hereditary	Precursor protein	Underlying disorder	Heart	Kidney liver	Liver	PN/AN	ST
AL	Acquired	Monoclonal immunoglobulin light chain	Plasma cell dyscrasia	+++	+++	++	+	++
hATTR	Hereditary	Abnormal TTR	Mutated TTR gene	+++	-	-	+++	-
wtATTR	Acquired	Normal TTR	-	+++	-	-	-	+
AA	Acquired	SAA	Inflammatory disorders	+ late	+++	+ late	+	-
ALECT2	Acquired	LECT2	Uncertain	-	+++	++	-	-
AGel	Hereditary	Abnormal gelsolin	Mutation in gelsolin gene	-	+	-	++ cranial	-
AB2M	Acquired or hereditary	AB2M	Long term dialysis	-	-	-	+	+
AApoA1	Hereditary	Abnormal ApoA1	Mutations in apolipoprotein A1 Gene	+	++	++	+	+ testis

*AL*, light chain amyloidosis; *hATTR*, hereditary transthyretin amyloidosis; *wtATTR*, wild-type transthyretin amyloidosis; *AA*, secondary amyloidosis; *ALECT2*: leukocyte chemotactic factor 2 amyloidosis; *AB2M*, beta-2-microglobulin amyloidosis; *AApoA1*, apolipoprotein A1 amyloidosis; *PN*, peripheral neuropathy; *AN*, autonomic neuropathy; *ST*, soft tissue; +++, very common; ++, common; +, less common; -, not reported

## Epidemiology

Acquired amyloidosis is more common than hereditary subtypes. AL is the most common, affecting 10–12 persons/million per year. Wild-type ATTR amyloidosis occurs predominately in men >70 years of age and primarily impacts the heart, although soft tissue involvement is often seen. Studies suggest up to 10–15% of older adults with heart failure may have unrecognized wtATTR [15]. A third form of acquired systemic amyloidosis, AA amyloidosis, occurs because of poorly controlled inflammatory disease and deposition of the acute-phase reactant serum amyloid A-protein. While rarely reported, at 1–2 cases/million per year, it is almost certainly underdiagnosed [16]. It most commonly affects the kidneys but can impact other organs in late stages.

**Fig. 1:**
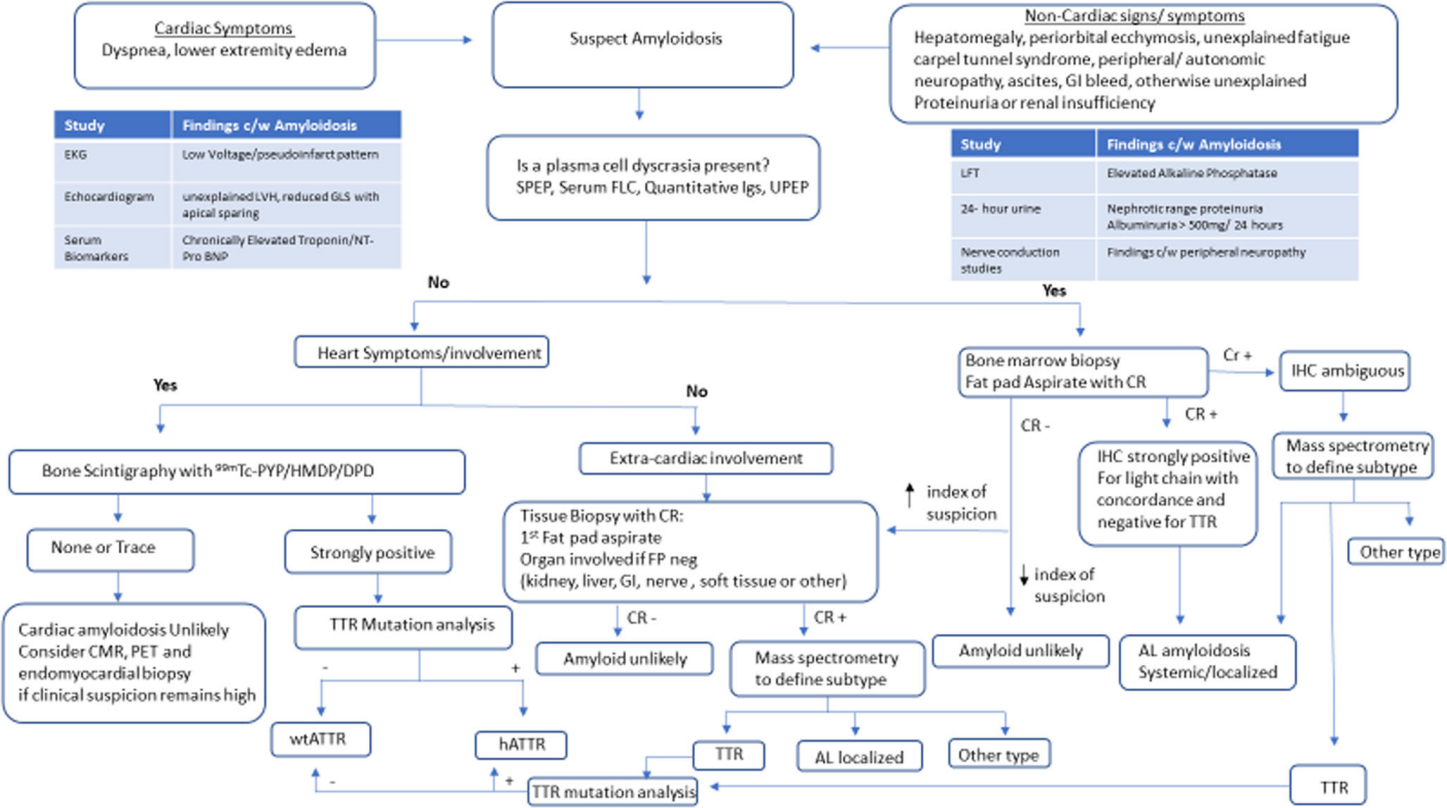
Diagnostic algorithm for systemic amyloidosis. EKG, electrocardiogram; Tpn, troponin; CMR, cardiac magnetic resonance; SPEP, serum protein electrophoresis; UPEP, urine protein electrophoresis; FLC, free light chains; Igs, immunoglobulins; BM, bone marrow; ^99m^Tc, ^99m^ technetium; PYP, pyrophosphate; DPD, 3,3-diphosphono-1,2-propanodicarboxylic acid; HMDP, hydroxymethylenediphosphonate; TTR, transthyretin; wtATTR, wild-type TTR amyloidosis; hATTR, hereditary TTR amyloidosis; IHC, immunohistochemistry, CR, Congo red

The most common form of hereditary amyloidosis results from a mutation of the TTR gene inherited in an autosomal dominant manner. More than 130 mutations of the TTR protein have been identified, the majority of which are noted to cause systemic amyloidosis, mainly impacting the peripheral nervous system and the heart [17]. Hereditary ATTR has a prevalence in the USA of 1 in 100,000 persons [18]. The most common mutation worldwide, which is associated with familial amyloid polyneuropathy (FAP), is the Val30Met variant found in patients of Portuguese, Swedish and Japanese decent. The most common mutation in the USA, the Val122Ile variant, is associated with ATTR cardiomyopathy and is carried by 3–4% of the African American population with variable penetrance [19]. In addition to TTR, there are other rarer forms of hereditary amyloidosis, including lysozyme and gelsolin amyloidosis. Leukocyte chemotactic factor 2 (LECT2) is the most recently identified form of systemic amyloidosis with predominant renal and hepatic involvement. This acquired form of amyloidosis primarily impacts the Hispanic population but has been described in South Asians as well. Appropriate workup and evaluation are critical in identifying the correct amyloid subtype to direct appropriate therapy (see Fig. 1).

## Cardiac Amyloidosis

Cardiac amyloidosis is the leading cause of morbidity and mortality in patients with systemic disease but remains underdiagnosed. In a recent study of 108 patients with heart failure with preserved ejection fraction (HFpEF), endomyocardial biopsy demonstrated that 14% of these patients had cardiac amyloidosis [20]. Patients with cardiac amyloidosis often present with non-specific heart failure symptoms including fatigue, dyspnea, decreased exercise tolerance, edema, and weight gain. Low blood pressure and intolerance of medications traditionally used to treat congestive heart failure are commonly encountered. The diagnosis of hypertrophic cardiomyopathy in an elderly adult should raise suspicion for amyloid-related heart disease. When cardiac amyloidosis is suspected, a detailed evaluation can define the subtype, thus guiding management (see Fig. 1).

Patients who may have cardiac amyloidosis should have a comprehensive evaluation with an EKG, echocardiogram, and laboratory evaluation including serum troponin as well as NT-ProBNP, biomarkers used for staging [21]. Because AL is the most common type of systemic amyloidosis in the developed world, all patients should undergo evaluation for a monoclonal gammopathy. Treatment and reduction of systemic free light chains in AL amyloidosis are generally followed by a reduction in NT-proBNP and troponin and consequent improvement in heart failure and long-term survival [22]. Although these biomarkers have been validated in AL amyloidosis, they play a role in risk stratification of patients with ATTR amyloidosis as well.

Whereas cardiac biomarkers may assist in the diagnosis and management of cardiac amyloidosis, they are highly non-specific. NT-proBNP levels rise with a multitude of causes of diastolic heart failure or volume overload, including renal disease. Troponin is a marker of myocardial injury or stress from elevated myocardial oxygen demand, thus also non-specific; therefore, the use of cardiac imaging becomes paramount in the diagnosis of amyloid heart disease. Primary modalities utilized include echocardiography, cardiac magnetic resonance (CMR) imaging, and radionuclide imaging.

**Fig. 2:**
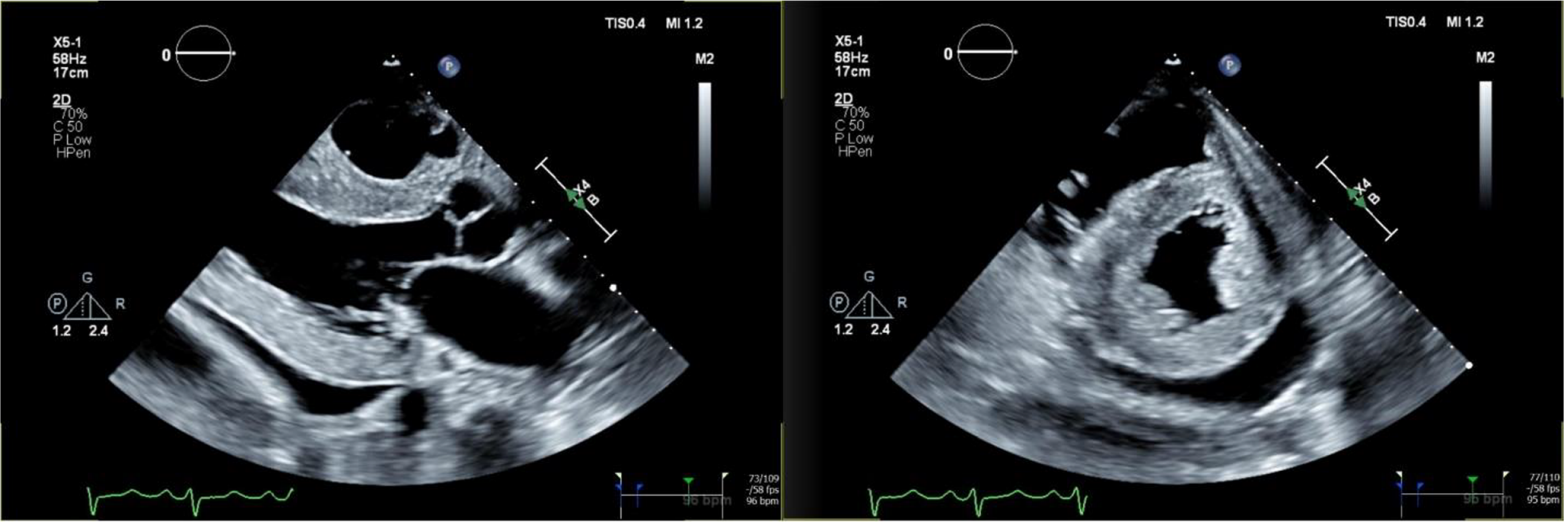
Parasternal long axis (left) and short axis (right) views of the heart demonstrate significant left ventricular hypertrophy with bright, sparkling left ventricular myocardium. A pericardial effusion is also evident posterior to the left ventricle

**Fig. 3:**
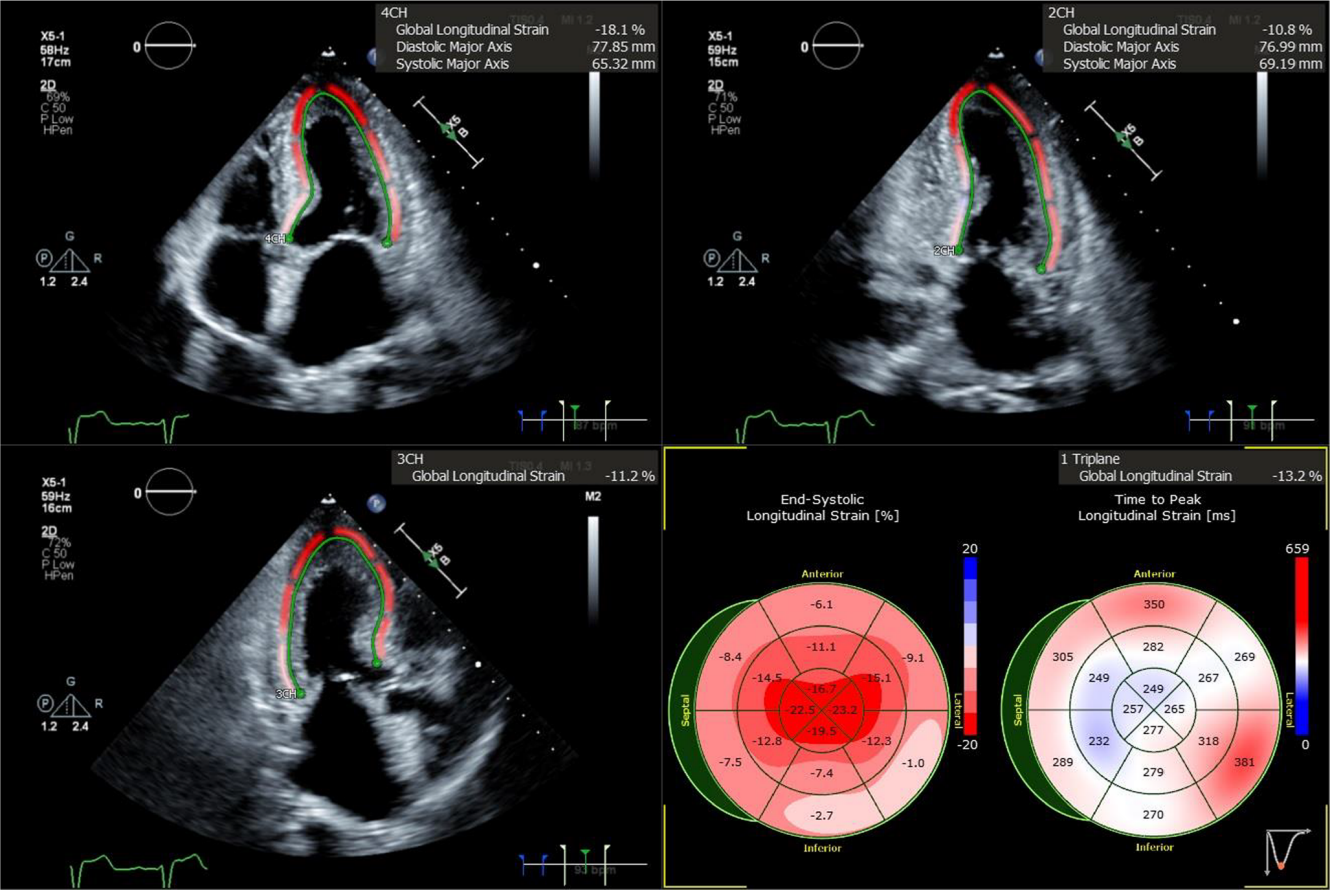
Three standard views of the heart are shown, as imaged from the LV apex. The green contours demonstrate calculation of global longitudinal strain (GLS). GLS values are color coded, with bright red reflecting normal longitudinal strain, while dark and lighter shades of pink indicate reductions in GLS. The apex of the heart is clearly spared, as red contouring is evident at the apical segments in all images, while strain is reduced in the basal and mid ventricular segments. The lower right quadrant shows a bull’s eye graph of the left ventricle divided into 16 segments; the preservation of healthy longitudinal strain at the apex results in a “cherry on top” pattern, pathognomonic for cardiac amyloidosis

Echocardiography in patients with cardiac amyloidosis often demonstrates left ventricular hypertrophy (LVH) with a sparkling appearance (see Fig. 2). Left ventricular function is gradually impacted as amyloid deposition within the myocardium progresses. Calculation of stroke volume generally demonstrates a reduction in affected patients. Speckle-tracking-derived myocardial strain imaging has emerged as a sensitive tool for evaluation of left ventricular function. We find reduction in global longitudinal strain to be a marker of early systolic dysfunction, while left ventricular ejection fraction often remains preserved until late stages. This clinical phenomenon is due to an earlier loss of systolic longitudinal contraction, whereas radial thickening and circumferential shortening remain preserved in early stages of disease. A particularly pathognomonic finding of amyloid heart disease is reduction in global longitudinal strain with sparing of the apex (see Fig. 3). This myocardial strain pattern has a sensitivity of 93% and specificity of 82% for distinguishing cardiac amyloidosis from other causes of LVH [23]. Other echocardiographic features often noted include thickening of the cardiac valves, right ventricular dysfunction, pericardial effusion, thickening of the interatrial septum, and dilatation of the atria. Atrial function is also impaired, and thrombus in the left atrium and left atrial appendage may occur in normal sinus rhythm, thus raising the risk of embolic stroke. Reduction of atrial contractility results in a restrictive mitral inflow pattern with a diminutive “a” wave on diastology. When heart failure is present, Doppler will demonstrate an elevation in left ventricular filling pressure, increased tricuspid valve regurgitation jet velocity, and dilatation of the inferior vena cava. Although there is no prognostic staging system based upon echocardiographic findings, these parameters are associated with worse outcomes and may be followed over time to evaluate for progression of disease as well as onset or worsening of heart failure [24•].

Electrocardiography may demonstrate low voltages and pseudoinfarct patterns [25•]. When combined with echocardiography, a classic finding of voltage-mass mismatch is often noted, which describes the discordance between the presence of LVH on echocardiography and low voltages on ECG. Although echocardiographic findings are generally similar between AL and ATTR types, a few differences have been described. LVH tends to be symmetric in AL type, whereas asymmetric, sigmoidal-shaped septal hypertrophy has been noted in ATTR [26]. ATTR is characterized by greater increases in left ventricular mass in comparison to right ventricular mass [27]. Furthermore, patients with wtATTR appear to have a greater increase in left ventricular mass and more reduction in LVEF [25•].

In addition to echocardiography, cardiac magnetic resonance (CMR) imaging is immensely valuable in aiding the diagnosis of cardiac amyloidosis. Unlike echocardiography, which has fairly limited tissue characterization power, CMR allows for detailed examination of the myocardial interstitium and evaluation of characteristic changes on late gadolinium enhancement (LGE) imaging, rendering CMR far more sensitive and specific than echocardiography [28]. Although CMR cannot distinguish between the AL vs ATTR phenotypes, it can assist in the early identification of cardiac amyloidosis before the presence of overt LVH [29]. Whereas echocardiography is limited to measurement of LV wall thickness, magnetic resonance imaging (MRI) can be utilized to calculate the extracellular volume within the myocardium. Classic LVH is characterized by myocyte hypertrophy; however, cardiac amyloidosis is caused by deposition of amyloid fibrils in the extracellular space, thus producing a marked increase in the extracellular volume. This change can be measured by T1 mapping, as myocardial T1 relaxation values correspond to the degree of underlying myocardial infiltration, edema, and fibrosis [24•]. Several differing patterns of enhancement have been reported on LGE imaging, but the most common distributions involve global subendocardial and transmural change [24•]. Subendocardial LGE has been found to be the predominant pattern in AL amyloidosis, whereas transmural LGE is more common in ATTR disease [30]. A typical LGE pattern has a sensitivity of 85 to 90% for the diagnosis of cardiac amyloidosis [24•]. LGE imaging can also be used to monitor progression of disease as amyloid infiltration progresses, thus allowing the LGE pattern to serve as an independent predictor of prognosis [31]. Although not well validated, the quantitative power of CMR has also been used to track response to chemotherapy by assessing for reduction in LV mass and extracellular volume [32].

Endomyocardial biopsy has been the gold standard for pathologic confirmation of cardiac amyloidosis, but radionuclide scintigraphy with ^99m^Technetium labeled bone-avid bisphosphonate derivatives has emerged as a highly sensitive and very specific technique to diagnose ATTR cardiac amyloidosis [24•]. Moreover, the degree of myocardial uptake correlates with overall mortality [33]. The three primary tracers in use include ^99m^Tc-labeled pyrophosphate (PYP), 3,3-diphosphono-1,2-propano-dicarboxylic acid (DPD), and hydroxymethylenediphosphonate (HMDP), with ^99m^Tc-PYP being the most commonly used tracer in the USA. It is unclear why there is greater uptake of ^99m^Tc-PYP in hearts afflicted with ATTR, but it has been postulated that this increased uptake may be due to a higher calcium content or the composition of amyloid fibrils in ATTR compared to other forms [34]. Patients exhibiting the presence of a positive ^99m^Tc-PYP scan without monoclonal proteins in the blood and urine may be diagnosed with ATTR without tissue confirmation. The specificity and positive predictive value of this modality is >98% [35•]. If a plasma cell dyscrasia is identified, then a biopsy for histologic diagnosis is still warranted, as up to 20% of patients with AL cardiac amyloidosis have been reported to have significant uptake on ^99m^Tc-PYP/DPD/HMDP scanning [35•]. An elegant and important study describes the ability to diagnose ATTR amyloidosis without tissue confirmation in select cases in which patients have heart failure and an echocardiogram or CMR at least suggestive of amyloidosis, as well as grade 2 or 3 cardiac uptake on a radionucleotide scan and absence of a detectable monoclonal protein by comprehensive testing [35•]. A diagnostic algorithm for patients with suspected amyloid cardiomyopathy is shown to help identify patients with ATTR and to direct appropriate genetic testing and management (see Fig. 1).

Positive emission tomography (PET) is emerging as another imaging modality that may prove useful in the identification of cardiac amyloid infiltration. With multiple tracers under development and investigation, PET utilizes radiotracers that directly bind to amyloid fibrils. ^11^C-Pittsburgh compound B (PIB) was developed for beta-amyloid imaging, but use is limited to sites with a cyclotron [24•]. ^18^F-florbetapir and ^18^F-florbetaben are two other commonly used PET radiotracers proven to distinguish cardiac amyloidosis from other causes of cardiac hypertrophy [36, 37].

Although development of various arrhythmias has been attributed to direct myocardial infiltration, this phenomenon may also be related to amyloid protein deposition impacting the cardiac innervation system. Autonomic dysfunction has been notably more common in patients with ATTR cardiac amyloidosis, particularly hATTR [38]. ^123^I-meta-iodobenzylguanidine (*m*IBG), a modified analogue of norepinephrine stored in presynaptic nerve terminals within the cardiac conduction system, has been used to image myocardial denervation [24•]. Although ^123^I-*m*IBG imaging is not helpful in diagnosing cardiac amyloidosis, it can be used to detect myocardial denervation earlier than detection of myocardial amyloid by ^99m^Tc-PYP/DPD/HMDP scanning in patients with an hATTR mutation [39].

The diagnosis of cardiac amyloidosis can be challenging and is often delayed. Cardiac imaging techniques as described are often neither sensitive nor specific, and the need for tissue biopsy with proper histological confirmation requires specialized centers and expertise. The diagnosis of wtATTR amyloidosis is particularly challenging because it presents at an older age often in patients with comorbidities. Furthermore, in contrast to hATTR and AL, there is an absence of supportive biomarkers in wild-type ATTR, including a TTR gene mutation or monoclonal gammopathy, respectively [40, 41]. With the advent of new treatments, there is a need to utilize multiple strategies and modalities to diagnose and characterize cardiac amyloidosis at the earliest opportunity.

## Diagnostic Evaluation

Making a diagnosis of amyloidosis must first begin with a clinical suspicion (see Fig. 1). Two features make this particularly challenging: the perceived rarity of the disorder and non-specific symptomatology. Patients with cardiac disease present with congestive heart failure, while patients with non-cardiac involvement present with a range of non-specific symptoms including sensory and autonomic neuropathy, lower extremity edema, diarrhea, constipation, early satiety, and abdominal distention with or without hepatomegaly. Symptoms as general as unexplained fatigue and weight loss may be early presenting symptoms of amyloidosis, particularly in the setting of a monoclonal gammopathy, and should therefore raise suspicion. When amyloidosis is suspected, workup should begin with a detailed cardiac examination and/or extra-cardiac evaluation. Because AL is the most common form of systemic disease and patients benefit from early recognition and treatment, all patients with a suspected diagnosis of amyloidosis should be evaluated for an underlying plasma cell dyscrasia.

**Fig. 4:**
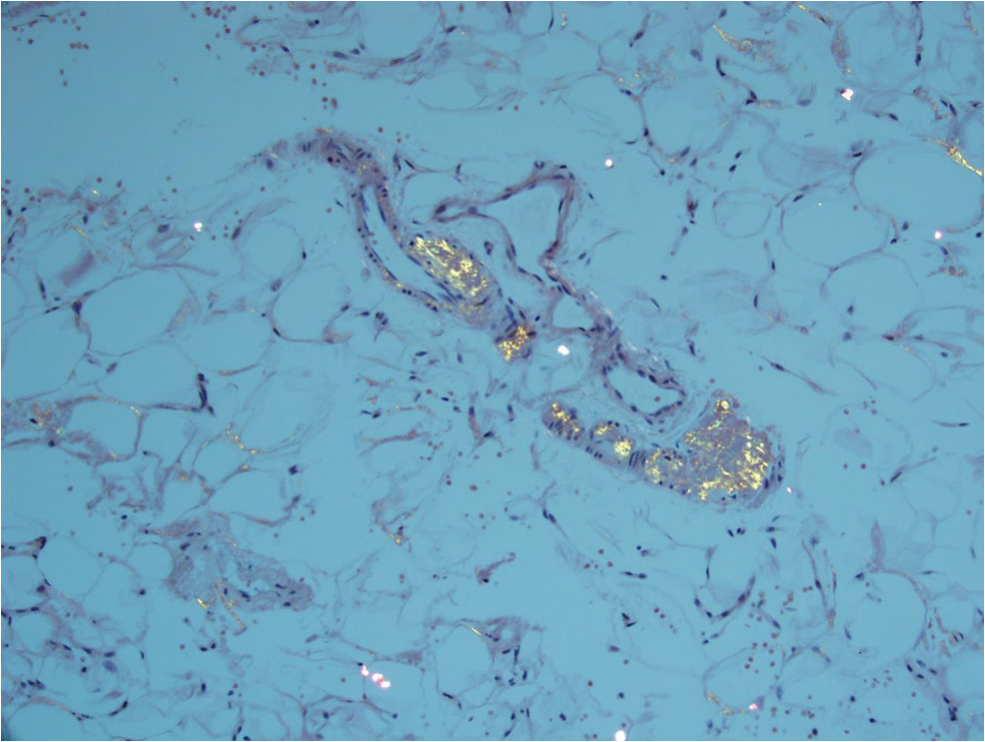
Fat pad aspiration sample with Congo red staining viewed under polarized light displaying green birefringence

Pathologic confirmation of amyloid deposition is the gold standard for diagnosing amyloidosis. In those with systemic disease, targeting the affected organ can be associated with complications; therefore, it is preferable to begin with a lower-risk screening procedure. An abdominal fat pad aspiration can be performed easily in the outpatient setting. With an overall sensitivity of 80%, the subcutaneous fat pad aspiration is the preferred method for detecting systemic amyloidosis [42]. It is important to recognize, however, that the sensitivity is highest in those with AL when compared to other forms of systemic disease. In a study of 216 patients with systemic amyloidosis, the sensitivity of the abdominal fat pad aspirate in AL, hATTR and wtATTR amyloidosis was 84%, 45%, and 15%, respectively [43]. If the fat pad aspirate fails to show amyloid deposition and suspicion remains high, a biopsy of an involved organ must be pursued. Pathologic demonstration of a fat pad aspirate positive for amyloid deposition is shown (see Fig. 4).

Once amyloid protein is identified, it is critical to correctly identify the precursor amyloidgenic protein and thus the pathologic subtype of disease. Immunohistochemistry or immunofluorescence can be used in this effort, but these techniques are often unreliable [44]. In AL, there should be concordance between the monoclonal light chain type noted by systemic plasma cell evaluation and the composition of amyloid fibrils. The leading method for identifying the amyloid subtype is laser microdissection mass spectrometry. Validation studies have demonstrated 100% sensitivity and specificity, which is far superior to other clinical and laboratory methods but requires tissues to be sent to specialized laboratories [45]. Treatment options for systemic amyloidosis vary considerably on the basis of the subtype; thus, clear understanding and definition of the disease is paramount to patient management.

## Treatment

Treatment options for patients with systemic amyloidosis have improved tremendously in recent years. In general, all treatments for systemic amyloidosis focus on targeting the underlying production of amyloidgenic protein. In AL amyloidosis, treatments have been adopted from those used in multiple myeloma to target the underlying plasma cell dyscrasia. For upfront management, selection of a treatment should deliver the most rapid and effective reduction in monoclonal protein that the patient can safely tolerate while taking into consideration severity of organ involvement, characteristics of the clone, and the patient’s comorbidities [46]. Early and deep reduction of the involved monoclonal, amyloidgenic protein is associated with the greatest chance of organ improvement and prolongation of progression-free and overall survival [47•, 48]. Changes in hematologic and organ-specific biomarkers have been validated to determine both hematologic and organ response to treatment [47•, 49–51].

Options for treatment of AL amyloidosis include traditional chemotherapy as well as high-dose melphalan, followed by autologous stem cell transplantation (ASCT). Careful selection of those eligible for high-dose therapy is critical in reducing treatment-related mortality, and only about 20% of patients are candidates for ASCT on initial presentation. More may become eligible after effective upfront therapy; however, in some patients, upfront chemotherapy may induce toxicity and render a once transplant-eligible patient subsequently ineligible. For those with excellent performance status and ≤10% plasma cell burden at presentation, proceeding directly to ASCT without any induction is an option [52], but induction chemotherapy prior to transplant has been shown to improve progression-free survival (PFS), such that 2–4 cycles of induction chemotherapy given prior to stem cell collection are reasonable to consider [53]. With appropriate patient selection at experienced centers, outcomes with transplantation can be superb. A report of 629 patients with AL amyloidosis who underwent transplantation at Boston University reported a median overall survival (OS) of 7.6 years. Importantly, the median OS was significantly better for those who achieved a complete response (CR), those without cardiac involvement and those with <2 organ systems involved. A long-term survival of >20 years occurred in 30% of patients [54].

For transplant-ineligible patients, melphalan and dexamethasone had been standard [55]; however, with the advent of novel, more targeted therapies, bortezomib-based induction regimens, which are generally well tolerated and efficacious, now form the backbone of conventional therapy [56]. In patients with amyloidosis, weekly dosing is better tolerated than twice weekly dosing, with less neurotoxicity [57]. A randomized phase 3 trial comparing melphalan and dexamethasone with or without bortezomib demonstrated improved hematologic responses of 81% with the triplet, in contrast to 57% when bortezomib was omitted. OS and organ response rates were higher as well when bortezomib was added [58]. The three-drug regimen of cyclophosphamide rather than melphalan in combination with bortezomib and dexamethasone (CyBorD) is more commonly used in the USA. CyBorD has resulted in high response rates (>90%) when used as upfront therapy, with 60% achieving at least a very good partial response (VGPR) [59, 60]. The ANDROMEDA study, a large phase 3 randomized trial, was designed to improve upon the standard of care by comparing CyBorD with or without the addition of the anti-CD38 monoclonal antibody daratumumab. The uncontrolled safety lead-in portion demonstrated an impressive hematologic response rate of 96% [61]. Preliminary results from the randomized trial show the four-drug combination to be well tolerated, with significantly higher hematologic (92 vs 77% overall; 79 vs 49% VGPR) cardiac (42 vs 22%) and renal (54 vs 27%) responses, leading to the first FDA-approved treatment for AL amyloidosis and suggesting a new standard for frontline treatment [62].

For patients with relapsed AL amyloidosis, there is no clear regimen of choice, and careful selection of anti-plasma cell strategies used for multiple myeloma should be considered on the basis of the burden of disease, prior exposures, and the patient’s comorbidities. Immunomodulatory drugs including lenalidomide and pomalidomide are effective but often tolerated at lower doses than those used for multiple myeloma [63, 64]. A phase 3 study of ixazomib, a first-in-class oral proteasome inhibitor, vs physician choice failed to meet its primary endpoint of improved overall hematologic response rate but did demonstrate improved CR and PFS compared to physicians’ choice [65]. Daratumumab has shown tremendous promise, with a phase 2 trial of single-agent daratumumab reporting a remarkable hematologic response rate of 90% (CR 41%) for patients with relapsed AL amyloidosis [66].

For those with ATTR amyloidosis, treatment is also focused on reducing the supply of precursor protein for amyloid fibril formation. Until recently, there were no FDA-approved medications to either reduce the concentration of amyloid fibrils or prevent misfolding of TTR and thus decrease amyloid formation and prevent further organ damage. The mainstay of treatment was liver transplantation. In 2018, two new small molecule oligonucleotides that inhibit the synthesis of TTR were approved for the treatment of hATTR amyloidosis in patients with a confirmed genetic mutation and peripheral neuropathy. Patisiran, a small molecule RNA interference agent, was compared to placebo in patients with hATTR and polyneuropathy. Subjects receiving patisiran had reduced levels of TTR as well as significantly improvement of peripheral neuropathy scores and quality of life [67]. A similar trial using an antisense oligonucleotide approach with inotersen also resulted in slowing of neuropathy progression [68]. An alternative approach to decreasing TTR concentration as management of ATTR is stabilization of the TTR tetramer. Randomized controlled trials of TTR tetramer stabilizers, tafamidis and diflunisal, have shown clinical efficacy and can be used for patients with either wtATTR or hATTR and neuropathy or cardiomyopathy [69, 70].

Anti-amyloid strategies to treat fibril deposition in damaged organs have unfortunately failed to yield favorable results. Efforts are continuing, however, and trials exploring doxycycline (NCT02207556) and an anti-fibril monoclonal antibody, CAEL-101 (NCT04512235, NCT04504825), are ongoing.

Although amyloidosis remains rare, both acquired and inherited subtypes are likely underdiagnosed. As new treatments become available and outcomes improve with the advent of novel approaches to target the production of amyloidgenic proteins, it is increasingly important for physicians to identify patients with amyloidosis and characterize the subtype accurately. Cardiac amyloidosis contributes significantly to the morbidity and mortality associated with systemic disease, and new tools are available to assist with diagnosis, prognosis, and management.

## References

[1] Falk RH, Comenzo RL, Skinner M (1997). The systemic amyloidoses. N Engl J Med.

[2] Bonar L, Cohen AS, Skinner MM (1969). Characterization of the amyloid fibril as a cross-beta protein. Proc Soc Exp Biol Med.

[3] Glenner GG, Terry WD (1974). Characterization of amyloid. Annu Rev Med.

[4] Pepys MB, Rademacher TW, Amatayakul-Chantler S, Williams P, Noble GE, Hutchinson WL, Hawkins PN, Nelson SR, Gallimore JR, Herbert J (1994). Human serum amyloid P component is an invariant constituent of amyloid deposits and has a uniquely homogeneous glycostructure. Proc Natl Acad Sci U S A.

[5] Tan SY, Pepys MB (1994). Amyloidosis. Histopathology.

[6] Wechalekar AD, Gillmore JD, Hawkins PN (2016). Systemic amyloidosis. Lancet.

[7] Cowan AJ, Skinner M, Seldin DC, Berk JL, Lichtenstein DR, O'Hara CJ, Doros G, Sanchorawala V (2013). Amyloidosis of the gastrointestinal tract: a 13-year, single-center, referral experience. Haematologica.

[8] Gertz MA, Comenzo R, Falk RH, Fermand JP, Hazenberg BP, Hawkins PN, Merlini G, Moreau P, Ronco P, Sanchorawala V, Sezer O, Solomon A, Grateau G (2005). Definition of organ involvement and treatment response in immunoglobulin light chain amyloidosis (AL): a consensus opinion from the 10th International Symposium on Amyloid and Amyloidosis, Tours, France, 18-22 April 2004. Am J Hematol.

[9] Basset M, Hummedah K, Kimmich C, Veelken K, Dittrich T, Brandelik S, Kreuter M, Hassel J, Bosch N, Stuhlmann-Laeisz C, Blank N, Müller-Tidow C, Röcken C, Hegenbart U, Schönland S (2020). Localized immunoglobulin light chain amyloidosis: novel insights including prognostic factors for local progression. Am J Hematol.

[10] Sipe JD, Benson MD, Buxbaum JN, Ikeda SI, Merlini G, Saraiva MJM, Westermark P (2014). Nomenclature 2014: amyloid fibril proteins and clinical classification of the amyloidosis. Amyloid.

[11] Falk RH (2005). Diagnosis and management of the cardiac amyloidoses. Circulation.

[12] Palladini G, Lavatelli F, Russo P, Perlini S, Perfetti V, Bosoni T, Obici L, Bradwell AR, D'Eril GVM, Fogari R, Moratti R, Merlini G (2006). Circulating amyloidogenic free light chains and serum N-terminal natriuretic peptide type B decrease simultaneously in association with improvement of survival in AL. Blood.

[13] Diomede L, Rognoni P, Lavatelli F, Romeo M, del Favero E, Cantù L, Ghibaudi E, di Fonzo A, Corbelli A, Fiordaliso F, Palladini G, Valentini V, Perfetti V, Salmona M, Merlini G (2014). A Caenorhabditis elegans-based assay recognizes immunoglobulin light chains causing heart amyloidosis. Blood.

[14] Sperry BW, Reyes BA, Ikram A, Donnelly JP, Phelan D, Jaber WA, Shapiro D, Evans PJ, Maschke S, Kilpatrick SE, Tan CD, Rodriguez ER, Monteiro C, Tang WHW, Kelly JW, Seitz WH, Hanna M (2018). Tenosynovial and cardiac amyloidosis in patients undergoing carpal tunnel release. J Am Coll Cardiol.

[15] Ruberg FL, Grogan M, Hanna M, Kelly JW, Maurer MS (2019). Transthyretin amyloid cardiomyopathy: JACC State-of-the-Art Review. J Am Coll Cardiol.

[16] Papa R, Lachmann HJ (2018). Secondary, AA, Amyloidosis. Rheum Dis Clin N Am.

[17] Adams D, Koike H, Slama M, Coelho T (2019). Hereditary transthyretin amyloidosis: a model of medical progress for a fatal disease. Nat Rev Neurol.

[18] Ando Y, Coelho T, Berk JL, Cruz MW, Ericzon BG, Ikeda SI, Lewis WD, Obici L, Planté-Bordeneuve V, Rapezzi C, Said G, Salvi F (2013). Guideline of transthyretin-related hereditary amyloidosis for clinicians. Orphanet J Rare Dis.

[19] Maurer MS, Hanna M, Grogan M, Dispenzieri A, Witteles R, Drachman B, Judge DP, Lenihan DJ, Gottlieb SS, Shah SJ, Steidley DE, Ventura H, Murali S, Silver MA, Jacoby D, Fedson S, Hummel SL, Kristen AV, Damy T, Planté-Bordeneuve V, Coelho T, Mundayat R, Suhr OB, Waddington Cruz M, Rapezzi C, THAOS Investigators (2016). Genotype and phenotype of transthyretin cardiac amyloidosis: THAOS (Transthyretin Amyloid Outcome Survey). J Am Coll Cardiol.

[20] Hahn VS, Yanek LR, Vaishnav J, Ying W, Vaidya D, Lee YZJ, Riley SJ, Subramanya V, Brown EE, Hopkins CD, Ononogbu S, Perzel Mandell K, Halushka MK, Steenbergen C, Rosenberg AZ, Tedford RJ, Judge DP, Shah SJ, Russell SD, Kass DA, Sharma K (2020). Endomyocardial biopsy characterization of heart failure with preserved ejection fraction and prevalence of cardiac amyloidosis. JACC Heart Fail.

[21] Kumar S, Dispenzieri A, Lacy MQ, Hayman SR, Buadi FK, Colby C, Laumann K, Zeldenrust SR, Leung N, Dingli D, Greipp PR, Lust JA, Russell SJ, Kyle RA, Rajkumar SV, Gertz MA (2012). Revised prognostic staging system for light chain amyloidosis incorporating cardiac biomarkers and serum free light chain measurements. J Clin Oncol.

[22] Merlini G, Lousada I, Ando Y, Dispenzieri A, Gertz MA, Grogan M, Maurer MS, Sanchorawala V, Wechalekar A, Palladini G, Comenzo RL (2016). Rationale, application and clinical qualification for NT-proBNP as a surrogate end point in pivotal clinical trials in patients with AL amyloidosis. Leukemia.

[23] Phelan D, Collier P, Thavendiranathan P, Popović ZB, Hanna M, Plana JC, Marwick TH, Thomas JD (2012). Relative apical sparing of longitudinal strain using two-dimensional speckle-tracking echocardiography is both sensitive and specific for the diagnosis of cardiac amyloidosis. Heart.

[24] • Dorbala S, et al. ASNC/AHA/ASE/EANM/HFSA/ISA/SCMR/SNMMI expert consensus recommendations for multimodality imaging in cardiac amyloidosis: part 1 of 2-evidence base and standardized methods of imaging. J Nucl Cardiol. 2019;26(6):2065–123. **This text provides guidelines for imaging in patients with suspected cardiac amyloidosis.** 10.1007/s12350-019-01760-6 31468376

[25] • Rapezzi C, et al. Systemic cardiac amyloidoses: disease profiles and clinical courses of the 3 main types. Circulation. 2009;120(13):1203–12. **Nuances in the various subtypes of cardiac amyloidosis are highlighted.** 10.1161/CIRCULATIONAHA.108.843334 19752327

[26] Martinez-Naharro A, Treibel TA, Abdel-Gadir A, Bulluck H, Zumbo G, Knight DS, Kotecha T, Francis R, Hutt DF, Rezk T, Rosmini S, Quarta CC, Whelan CJ, Kellman P, Gillmore JD, Moon JC, Hawkins PN, Fontana M (2017). Magnetic resonance in transthyretin cardiac amyloidosis. J Am Coll Cardiol.

[27] Maurer MS, Elliott P, Comenzo R, Semigran M, Rapezzi C (2017). Addressing common questions encountered in the diagnosis and management of cardiac amyloidosis. Circulation.

[28] Maceira AM, Joshi J, Prasad SK, Moon JC, Perugini E, Harding I, Sheppard MN, Poole-Wilson PA, Hawkins PN, Pennell DJ (2005). Cardiovascular magnetic resonance in cardiac amyloidosis. Circulation.

[29] Brownrigg J, Lorenzini M, Lumley M, Elliott P (2019). Diagnostic performance of imaging investigations in detecting and differentiating cardiac amyloidosis: a systematic review and meta-analysis. ESC Heart Fail.

[30] Fontana M, Pica S, Reant P, Abdel-Gadir A, Treibel TA, Banypersad SM, Maestrini V, Barcella W, Rosmini S, Bulluck H, Sayed RH, Patel K, Mamhood S, Bucciarelli-Ducci C, Whelan CJ, Herrey AS, Lachmann HJ, Wechalekar AD, Manisty CH, Schelbert EB, Kellman P, Gillmore JD, Hawkins PN, Moon JC (2015). Prognostic value of late gadolinium enhancement cardiovascular magnetic resonance in cardiac amyloidosis. Circulation.

[31] Raina S, Lensing SY, Nairooz RS, Pothineni NV, Hakeem A, Bhatti S, Pandey T (2016). Prognostic value of late gadolinium enhancement CMR in systemic amyloidosis. JACC Cardiovasc Imaging.

[32] Martinez-Naharro A, Abdel-Gadir A, Treibel TA, Zumbo G, Knight DS, Rosmini S, Lane T, Mahmood S, Sachchithanantham S, Whelan CJ, Lachmann HJ, Wechalekar AD, Kellman P, Gillmore JD, Moon JC, Hawkins PN, Fontana M (2018). CMR-verified regression of cardiac AL amyloid after chemotherapy. JACC Cardiovasc Imaging.

[33] Castano A, Haq M, Narotsky DL, Goldsmith J, Weinberg RL, Morgenstern R, Pozniakoff T, Ruberg FL, Miller EJ, Berk JL, Dispenzieri A, Grogan M, Johnson G, Bokhari S, Maurer MS (2016). Multicenter study of planar technetium 99m pyrophosphate cardiac imaging: predicting survival for patients with ATTR cardiac amyloidosis. JAMA Cardiol.

[34] Suhr OB, Lundgren E, Westermark P (2017). One mutation, two distinct disease variants: unravelling the impact of transthyretin amyloid fibril composition. J Intern Med.

[35] • Gillmore JD, et al. Nonbiopsy diagnosis of cardiac transthyretin amyloidosis. Circulation. 2016;133(24):2404–12. **This article illustrates the ability to identify cardiac transthyretin amyloidosis without biopsy.** 10.1161/CIRCULATIONAHA.116.021612 27143678

[36] Dorbala S, Vangala D, Semer J, Strader C, Bruyere JR, di Carli MF, Moore SC, Falk RH (2014). Imaging cardiac amyloidosis: a pilot study using (1)(8)F-florbetapir positron emission tomography. Eur J Nucl Med Mol Imaging.

[37] Law WP, Wang WYS, Moore PT, Mollee PN, Ng ACT (2016). Cardiac amyloid imaging with 18F-florbetaben PET: a pilot study. J Nucl Med.

[38] Delahaye N, Dinanian S, Slama MS, Mzabi H, Samuel D, Adams D, Merlet P, le Guludec D (1999). Cardiac sympathetic denervation in familial amyloid polyneuropathy assessed by iodine-123 metaiodobenzylguanidine scintigraphy and heart rate variability. Eur J Nucl Med.

[39] Piekarski E, Chequer R, Algalarrondo V, Eliahou L, Mahida B, Vigne J, Adams D, Slama MS, le Guludec D, Rouzet F (2018). Cardiac denervation evidenced by MIBG occurs earlier than amyloid deposits detection by diphosphonate scintigraphy in TTR mutation carriers. Eur J Nucl Med Mol Imaging.

[40] Rowczenio DM, Noor I, Gillmore JD, Lachmann HJ, Whelan C, Hawkins PN, Obici L, Westermark P, Grateau G, Wechalekar AD (2014). Online registry for mutations in hereditary amyloidosis including nomenclature recommendations. Hum Mutat.

[41] Bradwell AR, Carr-Smith HD, Mead GP, Tang LX, Showell PJ, Drayson MT, Drew R (2001). Highly sensitive, automated immunoassay for immunoglobulin free light chains in serum and urine. Clin Chem.

[42] Van Gameren II (2006). Diagnostic accuracy of subcutaneous abdominal fat tissue aspiration for detecting systemic amyloidosis and its utility in clinical practice. Arthritis Rheum.

[43] Quarta CC, Gonzalez-Lopez E, Gilbertson JA, Botcher N, Rowczenio D, Petrie A, Rezk T, Youngstein T, Mahmood S, Sachchithanantham S, Lachmann HJ, Fontana M, Whelan CJ, Wechalekar AD, Hawkins PN, Gillmore JD (2017). Diagnostic sensitivity of abdominal fat aspiration in cardiac amyloidosis. Eur Heart J.

[44] Solomon A, Murphy CL, Westermark P (2008). Unreliability of immunohistochemistry for typing amyloid deposits. Arch Pathol Lab Med.

[45] Dogan A (2017). Amyloidosis: insights from proteomics. Annu Rev Pathol.

[46] Palladini G, Milani P, Merlini G (2020). Management of AL amyloidosis in 2020. Blood.

[47] • Palladini G, et al. New criteria for response to treatment in immunoglobulin light chain amyloidosis based on free light chain measurement and cardiac biomarkers: impact on survival outcomes. J Clin Oncol. 2012;30(36):4541–9. **This manuscript demonstrates the importance of biomarkers and their utility in patient management.** 10.1200/JCO.2011.37.7614 23091105

[48] Muchtar E, Gertz MA, Lacy MQ, Leung N, Buadi FK, Dingli D, Hayman SR, Go RS, Kapoor P, Gonsalves W, Kourelis TV, Warsame R, Hwa YL, Fonder A, Hobbs M, Russell S, Lust JA, Siddiqui M, Rajkumar SV, Kyle RA, Kumar SK, Dispenzieri A (2020). Refining amyloid complete hematological response: quantitative serum free light chains superior to ratio. Am J Hematol.

[49] Palladini G, Hegenbart U, Milani P, Kimmich C, Foli A, Ho AD, Rosin MV, Albertini R, Moratti R, Merlini G, Schönland S (2014). A staging system for renal outcome and early markers of renal response to chemotherapy in AL amyloidosis. Blood.

[50] Sidana S, Milani P, Binder M, Basset M, Tandon N, Foli A, Dispenzieri A, Gertz MA, Hayman SR, Buadi FK, Lacy MQ, Kapoor P, Leung N, Rajkumar SV, Merlini G, Palladini G, Kumar SK (2020). A validated composite organ and hematologic response model for early assessment of treatment outcomes in light chain amyloidosis. Blood Cancer J.

[51] Lilleness B, Doros G, Ruberg FL, Sanchorawala V (2020). Establishment of brain natriuretic peptide - based criteria for evaluating cardiac response to treatment in light chain (AL) amyloidosis. Br J Haematol.

[52] Varga C, Comenzo RL (2019). High-dose melphalan and stem cell transplantation in systemic AL amyloidosis in the era of novel anti-plasma cell therapy: a comprehensive review. Bone Marrow Transplant.

[53] Cornell RF, et al. Bortezomib induction prior to autologous hematopoietic cell transplantation (AHCT) for newly diagnosed light chain amyloidosis (AL): A study of 426 patients. J Clin Oncol. 2020;38(15_suppl):8515–8515.

[54] Sanchorawala V, Sun F, Quillen K, Sloan JM, Berk JL, Seldin DC (2015). Long-term outcome of patients with AL amyloidosis treated with high-dose melphalan and stem cell transplantation: 20-year experience. Blood.

[55] Palladini G, Perfetti V, Obici L, Caccialanza R, Semino A, Adami F, Cavallero G, Rustichelli R, Virga G, Merlini G (2004). Association of melphalan and high-dose dexamethasone is effective and well tolerated in patients with AL (primary) amyloidosis who are ineligible for stem cell transplantation. Blood.

[56] Kastritis E, Anagnostopoulos A, Roussou M, Toumanidis S, Pamboukas C, Migkou M, Tassidou A, Xilouri I, Delibasi S, Psimenou E, Mellou S, Terpos E, Nanas J, Dimopoulos MA (2007). Treatment of light chain (AL) amyloidosis with the combination of bortezomib and dexamethasone. Haematologica.

[57] Gertz MA (2020). Immunoglobulin light chain amyloidosis: 2020 update on diagnosis, prognosis, and treatment. Am J Hematol.

[58] Kastritis E, Leleu X, Arnulf B, Zamagni E, Cibeira MT, Kwok F, Mollee P, Hájek R, Moreau P, Jaccard A, Schönland SO, Filshie R, Nicolas-Virelizier E, Augustson B, Mateos MV, Wechalekar A, Hachulla E, Milani P, Dimopoulos MA, Fermand JP, Foli A, Gavriatopoulou M, Klersy C, Palumbo A, Sonneveld P, Johnsen HE, Merlini G, Palladini G (2020). Bortezomib, melphalan, and dexamethasone for light-chain amyloidosis. J Clin Oncol.

[59] Venner CP, Lane T, Foard D, Rannigan L, Gibbs SDJ, Pinney JH, Whelan CJ, Lachmann HJ, Gillmore JD, Hawkins PN, Wechalekar AD (2012). Cyclophosphamide, bortezomib, and dexamethasone therapy in AL amyloidosis is associated with high clonal response rates and prolonged progression-free survival. Blood.

[60] Mikhael JR, Schuster SR, Jimenez-Zepeda VH, Bello N, Spong J, Reeder CB, Stewart AK, Bergsagel PL, Fonseca R (2012). Cyclophosphamide-bortezomib-dexamethasone (CyBorD) produces rapid and complete hematologic response in patients with AL amyloidosis. Blood.

[61] Palladini G, Kastritis E, Maurer MS, Zonder J, Minnema MC, Wechalekar AD, Jaccard A, Lee HC, Bumma N, Kaufman JL, Medvedova E, Kovacsovics T, Rosenzweig M, Sanchorawala V, Qin X, Vasey SY, Weiss BM, Vermeulen J, Merlini G, Comenzo RL (2020). Daratumumab plus CyBorD for patients with newly diagnosed AL amyloidosis: safety run-in results of ANDROMEDA. Blood.

[62] Kastritis, E., et al., Subcutaneous daratumumab + cyclophosphamide, bortezomib, and dexamethasone (CYBORD) in patients with newly diagnosed light chain (AL) amyloidosis: primary results from the phase 3 ANDROMEDA. EHA25 Virtual, Abstract LB2604, 2020.

[63] Dispenzieri A, Buadi F, Laumann K, LaPlant B, Hayman SR, Kumar SK, Dingli D, Zeldenrust SR, Mikhael JR, Hall R, Rajkumar SV, Reeder C, Fonseca R, Bergsagel PL, Stewart AK, Roy V, Witzig TE, Lust JA, Russell SJ, Gertz MA, Lacy MQ (2012). Activity of pomalidomide in patients with immunoglobulin light-chain amyloidosis. Blood.

[64] Sanchorawala V, Wright DG, Rosenzweig M, Finn KT, Fennessey S, Zeldis JB, Skinner M, Seldin DC (2007). Lenalidomide and dexamethasone in the treatment of AL amyloidosis: results of a phase 2 trial. Blood.

[65] Dispenzieri A, Kastritis E, Wechalekar AD, Schönland SO, Kim K, Sanchorawala V, Landau HJ, Kwok F, Suzuki K, Comenzo RL, Berg D, Liu G, Faller DV, Merlini G (2019). Primary results from the phase 3 Tourmaline-AL1 trial of ixazomib-dexamethasone versus physician’s choice of therapy in patients (Pts) with relapsed/refractory primary systemic AL amyloidosis (RRAL). Blood.

[66] Sanchorawala V, Sarosiek S, Schulman A, Mistark M, Migre ME, Cruz R, Sloan JM, Brauneis D, Shelton AC (2020). Safety, tolerability, and response rates of daratumumab in relapsed AL amyloidosis: results of a phase 2 study. Blood.

[67] Adams D, Gonzalez-Duarte A, O’Riordan WD, Yang CC, Ueda M, Kristen AV, Tournev I, Schmidt HH, Coelho T, Berk JL, Lin KP, Vita G, Attarian S, Planté-Bordeneuve V, Mezei MM, Campistol JM, Buades J, Brannagan TH, Kim BJ, Oh J, Parman Y, Sekijima Y, Hawkins PN, Solomon SD, Polydefkis M, Dyck PJ, Gandhi PJ, Goyal S, Chen J, Strahs AL, Nochur SV, Sweetser MT, Garg PP, Vaishnaw AK, Gollob JA, Suhr OB (2018). Patisiran, an RNAi therapeutic, for hereditary transthyretin amyloidosis. N Engl J Med.

[68] Benson MD, Waddington-Cruz M, Berk JL, Polydefkis M, Dyck PJ, Wang AK, Planté-Bordeneuve V, Barroso FA, Merlini G, Obici L, Scheinberg M, Brannagan TH, Litchy WJ, Whelan C, Drachman BM, Adams D, Heitner SB, Conceição I, Schmidt HH, Vita G, Campistol JM, Gamez J, Gorevic PD, Gane E, Shah AM, Solomon SD, Monia BP, Hughes SG, Kwoh TJ, McEvoy BW, Jung SW, Baker BF, Ackermann EJ, Gertz MA, Coelho T (2018). Inotersen treatment for patients with hereditary transthyretin amyloidosis. N Engl J Med.

[69] Berk JL, Suhr OB, Obici L, Sekijima Y, Zeldenrust SR, Yamashita T, Heneghan MA, Gorevic PD, Litchy WJ, Wiesman JF, Nordh E, Corato M, Lozza A, Cortese A, Robinson-Papp J, Colton T, Rybin DV, Bisbee AB, Ando Y, Ikeda S, Seldin DC, Merlini G, Skinner M, Kelly JW, Dyck PJ, Diflunisal Trial Consortium (2013). Repurposing diflunisal for familial amyloid polyneuropathy a randomized clinical trial. Jama-J Am Med Assoc.

[70] Coelho T, Maia LF, Martins da Silva A, Waddington Cruz M, Plante-Bordeneuve V, Lozeron P, Suhr OB, Campistol JM, Conceicao IM, Schmidt HHJ, Trigo P, Kelly JW, Labaudiniere R, Chan J, Packman J, Wilson A, Grogan DR (2012). Tafamidis for transthyretin familial amyloid polyneuropathy a randomized, controlled trial. Neurology.

